# Morphology-Based Deep Learning Approach for Predicting Osteogenic Differentiation

**DOI:** 10.3389/fbioe.2021.802794

**Published:** 2022-01-27

**Authors:** Yiqing Lan, Nannan Huang, Yiru Fu, Kehao Liu, He Zhang, Yuzhou Li, Sheng Yang

**Affiliations:** ^1^ Stomatological Hospital of Chongqing Medical University, Chongqing, China; ^2^ Chongqing Key Laboratory of Oral Diseases and Biomedical Sciences, Chongqing, China; ^3^ Chongqing Municipal Key Laboratory of Oral Biomedical Engineering of Higher Education, Chongqing, China

**Keywords:** deep learning, convolutional neural network, osteogenic differentiation, drug screening, online learning

## Abstract

Early, high-throughput, and accurate recognition of osteogenic differentiation of stem cells is urgently required in stem cell therapy, tissue engineering, and regenerative medicine. In this study, we established an automatic deep learning algorithm, i.e., osteogenic convolutional neural network (OCNN), to quantitatively measure the osteogenic differentiation of rat bone marrow mesenchymal stem cells (rBMSCs). rBMSCs stained with F-actin and DAPI during early differentiation (day 0, 1, 4, and 7) were captured using laser confocal scanning microscopy to train OCNN. As a result, OCNN successfully distinguished differentiated cells at a very early stage (24 h) with a high area under the curve (AUC) (0.94 ± 0.04) and correlated with conventional biochemical markers. Meanwhile, OCNN exhibited better prediction performance compared with the single morphological parameters and support vector machine. Furthermore, OCNN successfully predicted the dose-dependent effects of small-molecule osteogenic drugs and a cytokine. OCNN-based online learning models can further recognize the osteogenic differentiation of rBMSCs cultured on several material surfaces. Hence, this study initially demonstrated the foreground of OCNN in osteogenic drug and biomaterial screening for next-generation tissue engineering and stem cell research.

## Introduction

BMSCs are the most frequently used subtype of stem cells with a vigorous proliferative and differential capacity, making them a promising tool in tissue engineering, biomedicine, biomaterials, and many other fields (Mauney et al., 2005; Guan et al., 2012; Chiu et al., 2014; Yang et al., 2017; Farokhi et al., 2018; Qi et al., 2020). Assessing the osteogenic differentiation of BMSCs is of great importance for these applications, but is challenging because of the time-consuming process and low temporal-spatial resolution of conventional methods. For example, polymerase chain reaction and western blot only assess the bulk expression level, while histochemical staining, such as Alkaline phosphatase staining (ALP) and Alizarin red staining (ARS), often requires 14 days, 28 days, or even longer to induce observable biochemical changes (Waisman et al., 2019), which hinders the high-throughput screening of small molecules, cytokines, and biomaterials. Hence, an accurate, early-stage, and single-cell resolution method is urgently required to assess the osteogenic differentiation of BMSCs for next-generation biomedical applications.

During osteogenic differentiation, BMSCs tend to change from a spindle-like shape to a polygon-like shape and are enlarged *in vitro* (Fan et al., 2012), while the arrangement or texture of the cytoskeleton also manifests distinct alterations (Treiser et al., 2010; Oei et al., 2019). On the one hand, the osteogenesis process is accompanied by augmented cell volume and programmed cytoskeleton remodeling. On the other hand, the direct modulation of cellular adhesive areas and cytoskeletal texture substantially influence osteogenic differentiation (McBeath et al., 2004; Engler et al., 2006; Zhang et al., 2015). For instance, our previous studies demonstrated that the regulation of the cytoskeleton by surface topography directly mediates cell differentiation and is associated with the activation of multiple adhesion and morphological proteins, such as FAK, RhoA, and YAP (Zhang et al., 2015; Zhang et al., 2016; Li et al., 2019). Therefore, the cellular morphology of BMSCs provides invaluable information for osteogenic differentiation prediction (Thomas et al., 2002; Marklein et al., 2016).

The cellular morphology data comprise a large number of high-dimensional image features and are challenging for prediction methods. Previously, machine learning models, including the Bayesian linear regression (Cutiongco et al., 2020) and support vector machine (SVM) (Chen et al., 2016; Yelin et al., 2019; Chen et al., 2021), were successfully applied to predict early-stage cell osteogenic differentiation based on the cytoskeletal morphology of biomaterials with different micro-environmental cues. Nonetheless, the data processing and parameter optimization of conventional machine learning require a high degree of specialized knowledge and considerable human efforts. Fortunately, novel deep learning methods can avoid these manual processes and achieve a high performance (Moen et al., 2019), which has been successfully applied to predict cellular senescence (Kusumoto et al., 2021), neural stem cell differentiation (Zhu et al., 2021), and screening drugs (X. Yang et al., 2019).

**FIGURE 1:**
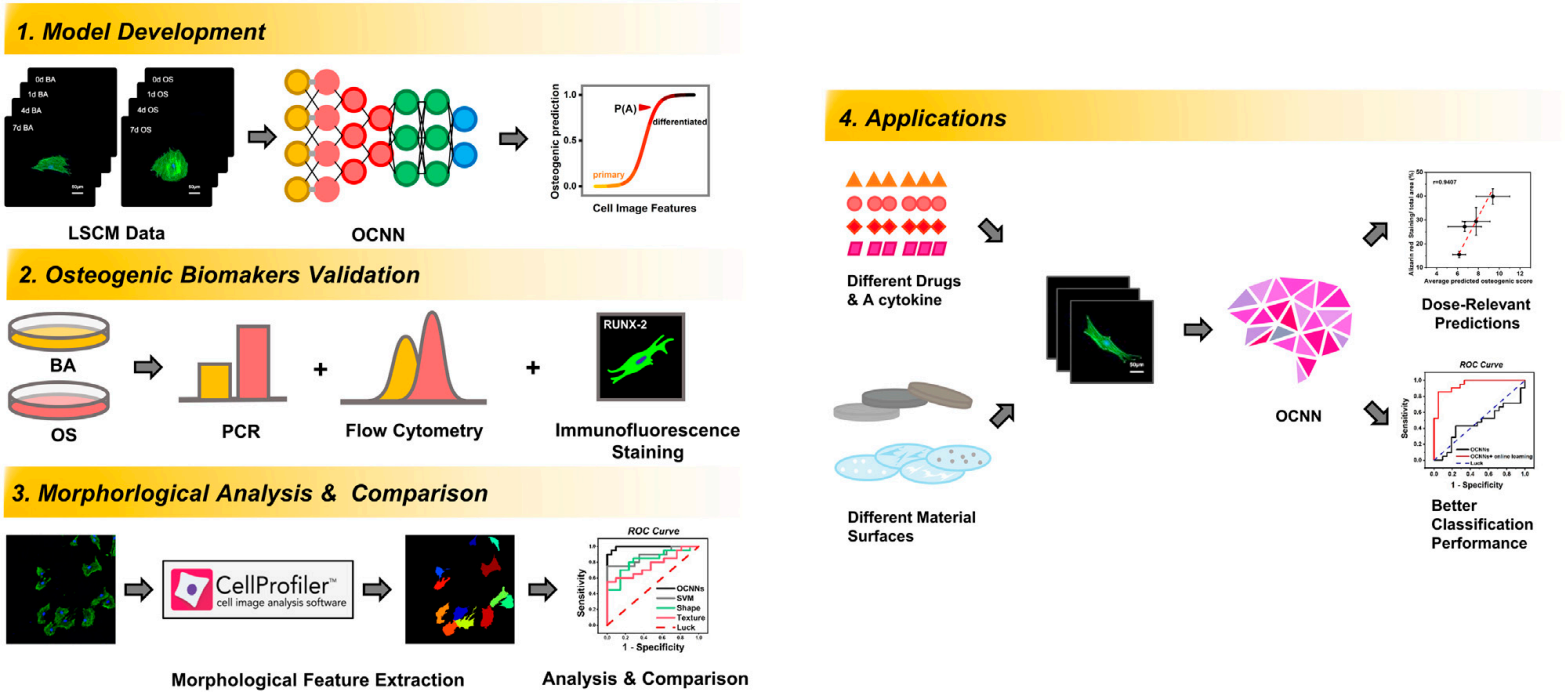
Schematic flow chart of the training, validation, comparison, and application procedures and outcomes of OCNN in this study.

Herein, as illustrated in Figure 1, we established and trained a deep learning model, called osteogenic convolutional neural network (OCNN), on the high-content laser scanning confocal microscope (LSCM) images of the rBMSCs during the early stages of osteogenic differentiation. The predicted osteogenic scores (POS) obtained from OCNN were verified using traditional biomarkers. Subsequently, we compared the performance of OCNN with single-cell morphological parameters and support vector machine models. Lastly, we evaluated the performance of the OCNN and its modification on the osteogenic differentiation predictions of the rBMSCs cultured with different soluble drugs and on different biomaterial substrates. We hope that this study could preliminarily demonstrate the promising potential of deep learning in stem cell research, drug screening, and novel biomaterial development.

## Materials and Methods

### Fabrication of Materials and Characterization of Surface Topography

Titanium plates with different nano-topographies were fabricated via sandblasting and acid etching, as previously described in our work (Li et al., 2019). Pure titanium plates (diameter of 14 mm and thickness of 1 mm, Chongqing University, Chongqing, China) were polished to 600 grit. Smooth surfaces were treated with 30 wt% HNO3 for 5 min. Micro surfaces were created by blasting with 100 l m aluminum oxide particles and incubated in 5 N HCl for 12 h and 30 wt% HNO3 for 5 min. Nano surfaces were manufactured *via* treatment with a 50/50 v/v% solution of 30% H_2_O_2_ and 2 N H_2_SO_4_ for 2 h. Titanium plates were ultrasonically washed with dd water and sterilized with 75 wt% ethanol and ultraviolet light.

The substrates were coated with hyaluronic acid (HA), collagen I (Col-I), poly-dopamine (DOPA), and amyloid fibrils as previously described briefly. The glass coverslips were cleaned ultrasonically in ethanol and then rinsed with deionized water. After air-drying and UV irradiation for 30 min, 1 ml aqueous solution of HA (1 mg/ml), Col-I (0.1 mg/ml, PBS), DOPA (2 mg/ml, 10 mM Tris-HCl, pH 8.5), and lysozyme (1 mg/ml, 50 mM TCEP) were poured onto the substrate and allowed to react for 3 days, 3, 16, and 2 h, respectively. The topography of the prepared titanium plates and glass coverslips with different surface coatings was observed using high-resolution scanning electron microscopy (SEM, Hitachi S-4700).

### Cell Culture

The rBMSCs were collected from the bone marrow of 4–6-week-old female rats as previously described (Ren et al., 2021) and identified by flow cytometry and differentiation phenotypes (Supplementary Figure S1). The cells were resuspended in complete medium and transferred to a Petri dish, cultured at 37°C with 5% CO_2_. The medium was changed after 1 day and 2 days respectively. After achieving confluency of 80–90%, the rBMSCs were digested, counted, and seeded on substrates with an initial cell density of 5,000–10,000 cells/cm^2^. In addition, they were cultured with Dulbecco’s Modified Eagle’s medium-low glucose containing 10% FBS and 1% penicillin/streptomycin and supplemented with PBS (basic medium, BA) or 10 nM dexamethasone, 10 mM β-glycerophosphate, and 50 μM ascorbic acid (osteogenic medium, OS). For the glass coverslips, the rBMSCs were cultured for 0, 1, 4, and 7 days. Whereas for the drug screening assays, they were cultured for 1 and 14 days, and for the biomaterial assays, they were cultured for 1 day. All animal operations were performed in accordance with the guidelines of the Animal Care and Use Committee of China and were approved by the ethics committee of Chongqing Medical University Affiliated Hospital of Stomatology (Ethic No. 2021033).

### Data Acquisition and Pre-processing

The rBMSCs were washed with PBS three times, fixed with 4% formaldehyde in Dulbecco’s phosphate-buffered saline (D-PBS), and washed repeatedly. Subsequently, they were permeabilized in 0.2% Triton-X100 (PBS) for 5 min and washed with PBS three times. Next, they were soaked in staining buffer containing 0.33 mM Alexa Fluor 488 phalloidin (Yisheng, China) and 10 mg/ml bovine serum albumin (PBS) for 1 h. After washing three times with PBS, nuclei were stained with 0.3 mМ 40, 6-diamidino-2-phenylindole (DAPI, Beyotime, China) for 3 min and then rinsed with PBS. Samples were observed and imaged with LSCM at a magnification of ×200 (Leica TCS SP8, Germany), and each 2-D image was taken at the maximum projection of the z-stack and saved as a 1,024 × 1024-pixel RGB image in the Tiff format.

For model development, single-cell images were cropped from the original LSCM images (5–10 cells per image) to obtain 2,916 single-cell images, approximately 500–600 images in each group (0, 1, 4, and 7 days with BA/OS). Notably, cells adjunct to other cells were abandoned because of the potential influence of cell-to-cell contact (Chen et al., 2016). The fluorescence intensity was globally normalized to correct the batch effect from different biological repeats.

### rBMSC Osteogenic Differentiation Assays

An mRNA was extracted by lysing the cells with TRIzol (Takara, Japan) and incubated with chloroform for 10 min, followed by centrifugation at 12,000 rpm for 15 min at 4°C. Then, it was purified using ethanol, resuspended in DEPC water (Biosharp, Japan), and quantified using a Nanodrop spectrophotometer (Thermo Scientific, Waltham, United States). Next, it was reverse transcribed to cDNA by using the RNAiso Plus reagent kit (Takara) and amplified using the ProFlex PCR system (Thermo Scientific). Finally, the genes expression levels were quantified using the Power SYBR Green PCR master mix (Takara) in a real-time PCR machine (Applied Biosystems 7500, Life Technologies, Waltham, United States). The primer sequences used in this study are listed in Supplementary Table S1.

For flow cytometry, the rBMSCs were digested, diluted with PBS, and stained with FITC Mouse Anti-Rat CD90 (Biolegend, San Diego, United States) and FITC Mouse Anti-Rat CD44 (Biolegend).

For immunofluorescence staining, they were fixed with 4% formaldehyde, permeabilized with 0.2% Triton-X100, and washed repeatedly. Then, they were blocked using 3% donkey serum for 1 h and incubated overnight with a Runx-2 primary antibody (1:200, Abcam, United Kingdom). After washing with PBS three times, cells were incubated with the fluorescent secondary antibody (1:350, Alexa 647, Jackson ImmunoResearch, West Grove, United States) for 1 h. Finally, the cytoskeleton and nuclei were stained according to the above-mentioned procedure.

For drug screening, the rBMSCs were treated with an OS medium accompanied by incremental concentrations of alendronate sodium (1, 5, or 10 μM), simvastatin (0.1 μg/ml, 0.5 μg/ml or 1 μg/ml), 1α, 25-dihydroxyvitamin D3 (1, 10 or 100 nM) as well as BMP-2 (50 ng/ml, 100 ng/ml and 200 ng/ml). After cultivation for 14 days, the cells were fixed with 4% paraformaldehyde for 10 min and washed with PBS. Then, they were stained with 1% Alizarin Red S (Solarbio) for 10 min and washed twice with PBS. The stained cells were captured using a stereomicroscope (Zeiss SteREO Discovery.V12, Germany) and quantified using ImageJ (NIH Image, Bethesda, MD).

### Development of Convolutional Neural Networks

To develop OCNN, three classic deep learning models with pre-trained weights (ImageNet), including VGG 16 (Simonyan and Zisserman, 2014), Inception V3 (Szegedy et al., 2016), and ResNet50 (He et al., 2016), were screened to select the convolutional core. The transfer learning method was used to extract the rBMSC features, and a binary classifier with softmax activation was added to the top of the models for predictions. The training process was conducted on a computer with an Intel Core i7-9700F, 32 GB RAM, and an NVIDIA GeForce RTX 2080Ti and implemented with Keras v.2.2.4 (http://github.com/fchollet/keras). Detailed information about the training of convolutional neural networks can be found in Supplementary Table S2.

During training, single-cell images were randomly distributed into training and validation sets at a ratio of 9:1. Conventional data augmentation was performed to reduce overfitting (Supplementary Figure S2). The Adam optimizer was adopted, and hyper-parameters (dropout ratio, learning rate, and batch size) were optimized to improve the performance. Ten-fold cross-validation was used to evaluate the prediction performance of the three models. For online learning, OCNN was further supplemented with small training samples (50–100) from different biomaterials.

To quantitatively measure the osteogenic differentiation in a linear space, predicted osteogenic score (POS) was proposed by a logit transformation of the final model output *p* for data scaling, similarly to a previously described method to quantify the cell senescence (Kusumoto et al., 2021):

$$POS=\;\ln\frac{p}{1-p}$$

### Single-Cell Morphological Parameters and Support Vector Machines

Cell morphology was measured on single-cell images using an open-source software Cellprofiler (the Broad Institute of Harvard and MIT, United States) (Lamprecht et al., 2007), yielding 25 single-cell morphological parameters. Six representative morphological parameters were selected by correlation analysis, including three shape parameters (area, perimeter, aspect ratio) and three texture parameters (contrast, correlation, and entropy).

Support vector machines were constructed using the Sci-kit package in Python. Feature dimensions were reduced to three from the morphological parameters using the linear kernel support vector machine technique. Hyperparameter optimization was conducted using the wrapping algorithm.

### Evaluation of Model Performance

To evaluate the model performance, the true positive (TP), true negative (TN), false positive (FP), and false negative (FN) values were counted. Then, six measurements, including accuracy, sensitivity, specificity, precision, recall, and F1-score, were calculated as follows:

$$Sensitivity=\frac{TP}{TP+FN}$$

$$Specificity=\frac{TN}{TN+FP}$$

$$Accuracy=\frac{TP+TN}{TP+FP+TN+FN}$$

$$\mathrm{Pr}ecision=\frac{TP}{TP+FP}$$

F1-score=2∗Precision∗RecallPrecision+Recall

The ROC curve was plotted based on the sensitivity and 1—Specificity scores, and the AUC value was computed.

### Statistical Analysis

The statistical charts were created using Origin 2018 (OriginLab, Northampton, United States), and the data were presented as mean ± standard deviations. For comparison between two groups, the unpaired Student’s t-test was used, and a value of <0.05 was considered statistically significant. For correlation analysis, a value of <0.05 was considered statistically significant.

## Results

### Morphological Characteristics of rBMSCs Under LSCM

**FIGURE 2:**
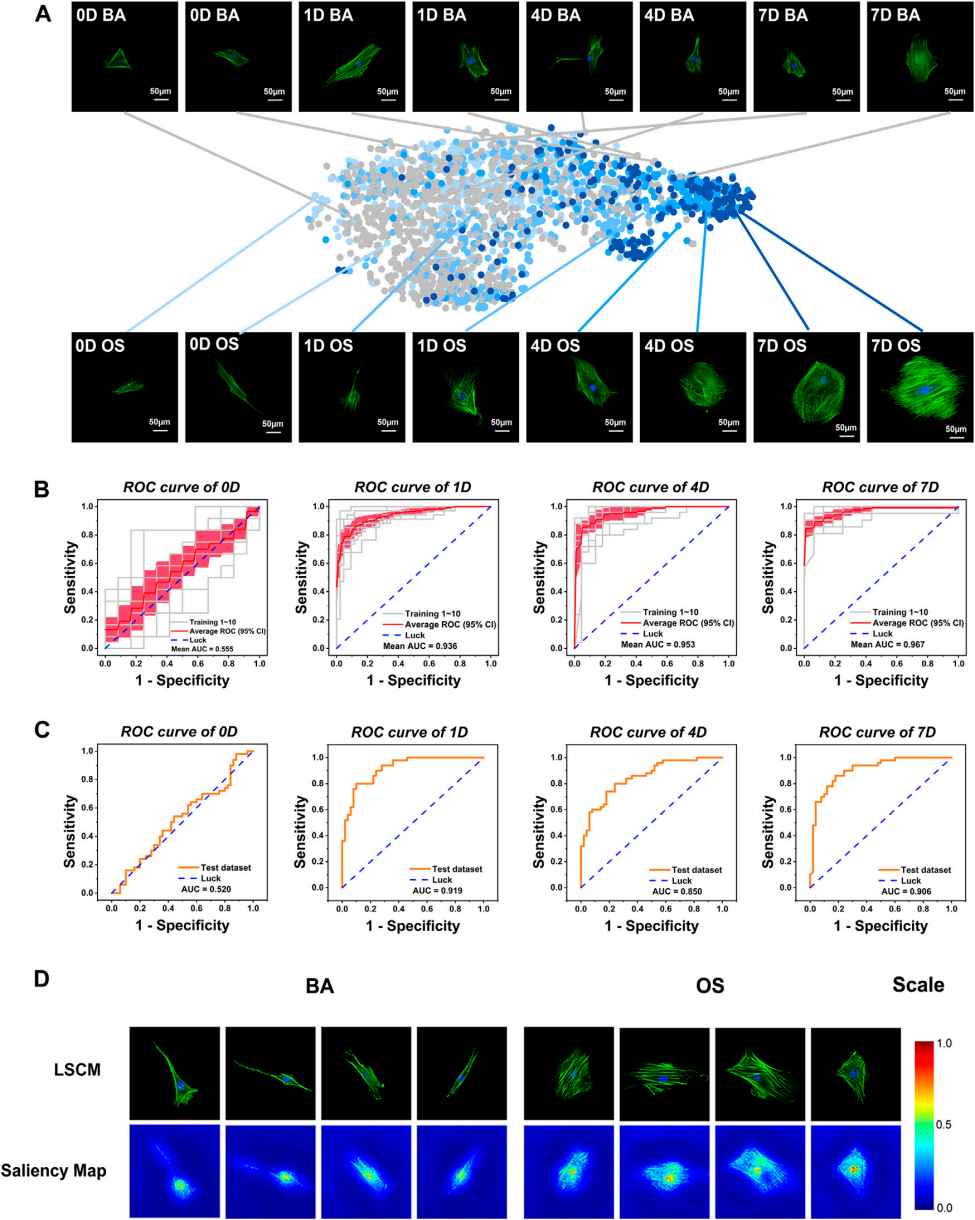
Development and validation of a conventional, static, and baseline model (OCNN) for sensitive high-throughput and automatic osteogenesis prediction on flat coverslips. **(A)** Visualization of diverse single-cell datasets. The styles from the network for all images in the cell dataset were embedded using t-SNE. Each point represents a different LSCM image. Grey: basic (BA) group; light to deep blue: osteogenic (OS) group on days 0,1,4, and 7. Each photo: green, F-actin; blue, nuclei. Scale bar, 50 µm. **(B)** 10-fold cross-validated ROC curves of Inception V3 at four-time points (validation dataset). The gray line represents each independent validation; the red line represents the average ROC; the light red area represents the 95% confidence interval. **(C)** A randomly selected new test dataset was used to evaluate the classification performance of OCNN (50 images of BA/OS group each). ROC curves at four-time points. The orange line represents the independent validation. **(D)** The saliency map showed key identification regions for the prediction of BA or OS cells.

To investigate the morphological changes of the rBMSCs during the early stages of osteogenic differentiation, we collected the rBMSCs cultured in osteogenic (OS) and basal (BA) mediums and then took LSCM images on days 0, 1, 4, and 7. As shown in Figure 2A, *via* the naked eye observation, the cellular shape changed from spindle-like to more extensive, and the cytoskeleton arrangement seemed to become more complex and crossed after induction for 4 days. However, by depositing 25 objective morphological parameters into two-dimensional t-SNE (Van der Maaten et al., 2008), we found a clear left-to-right shift of group OS starting from day 1, while group BA exhibited a more randomized distribution, which partially overlapped with the OS group.

To recognize the distinct but overlapping underlying pattern of the cellular morphology, we next developed OCNN models via the transfer learning of single-cell images using three classical deep learning models, VGG-16, Inception V3, and ResNet-50, as shown in Supplementary Figure S3 and Supplementary Table S3. Based on the general performance, we selected the pre-trained Inception V3 as the convolutional core of the OCNN to perform our follow-up studies. As shown in Figure 2B, the OCNN showed average AUCs of 0.936, 0.953, and 0.967 on days 1, 4, and 7, respectively, in 10-fold internal cross-validation. To validate the model generalization ability, OCNN was further tested on an independent, external dataset from biological repeats at four-time points, which also showed satisfactory AUCs of 0.919, 0.850, and 0.906 on days 1, 4, and 7, respectively (Figure 2C). The AUCs on day 0 were 0.555 (internal validation) and 0.520 (external validation), which is expected, since day 0 implies that cells had not received any treatment.

To better understand the morphological differences recognized by OCNN, we visualized the important regions that are relevant to the predictions via a Saliency Map (Simonyan, Vedaldi, et al., 2014), which reflected the activation of specific pixels upon specific predictions. Interestingly, as shown in Figure 2D, the nucleus and peri-nucleus cytoskeleton were mostly activated for the BA predictions, while more cytoplasmic cytoskeletons were activated in the OS prediction, probably reflecting the importance of the cytoskeleton in stemness or osteogenic differentiation. Nuclear morphology is an important indicator of cell function and is correlated with osteogenic differentiation via lamin A/C under external forces, nano-topography, and chemical coatings (Werner et al., 2017). On the other hand, the strong stress fibers of the cytoskeleton drew considerable concern in the OS group, as revealed in previous findings (Engler et al., 2006) that the intensity and arrangement of F-actin and non-muscle myosin-II plays an important role in MSC osteogenic differentiation. Considered together, these data showed the plausibility of the binary prediction models based on deep learning trained via nucleus and cytoskeleton morphology images.

It was worth noting that a small proportion of the images were incorrectly classified when using the OCNN, indicating that there may be some degree of differences in the level of single-cell differentiation within the stem cell population under nonosteogenic and osteogenic induction conditions, which cannot be classified via deep learning (Supplementary Figure S4).

### Biochemical Changes and Morphology-Based Predictions During Osteogenic Differentiation Compared with OCNN

Next, we analyzed the consistency between OCNN predictions and conventional biochemical measurements, including qRT-PCR, flow cytometry, and immunofluorescence staining. To compare in a one-dimensional linear space, we came up with the concept of the predicted osteogenic score (POS), a logit transformation value from the OCNN output for each single-cell image, as a modified method from previous studies.

**FIGURE 3:**
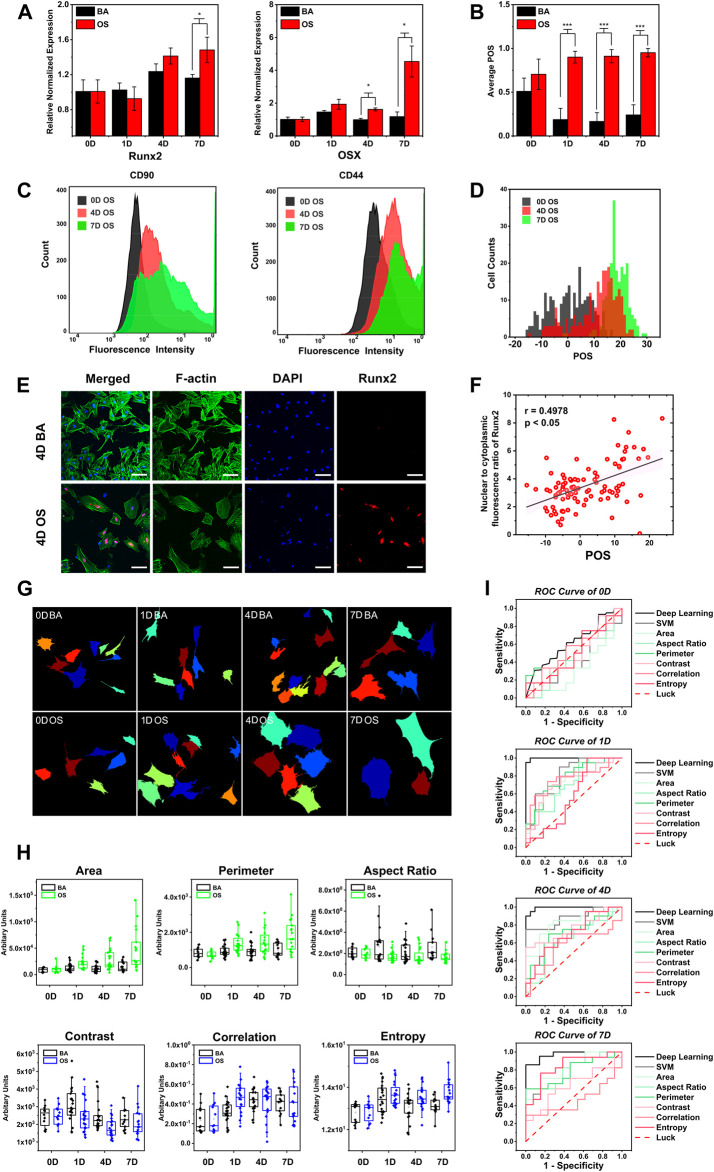
OCNN highly correlated with conventional biochemical markers and performed better than single morphological parameter and support vector machine in osteogenic differentiation prediction. **(A)** Real-time PCR gene expression levels of *Osx*, *Runx2* relative to *GAPDH* in rBMSCs cultured in BA/OS for 0, 1, 4, and 7 days **(B)** BA/OS Images of 0, 1, 4, and 7 days were classified and scored according to OCNN. **(C)** Flow cytometry determination of the MSC-specific surface markers (CD44, CD90) in OS groups at different induction times (0, 4, 7 days). **(D)** Images in the OS group of 0, 4, and 7 days were classified and scored according to OCNN. **(E)** rBMSCs’ immunofluorescence staining of Runx2 protein cultured in both types of the medium on day 4. Green: F-actin; Blue: Nuclei; Red: Runx2 protein. Scale bar: 100 µm. **(F)** Using the Nuclear/cytoplasm intensity ratio of Runx2 protein to define the extent of single-cell osteogenic differentiation and compared with OCNN predicting score on day 4. Data are shown as mean. *p* values by two-sided Student’s t-test. *: *p* < 0.05; ***: *p* < 0.001. **(G)** Schematic diagram of Cellprofiler software for cell localization and cell morphology capture. **(H)** Cell morphology was measured on the cell images of the validation dataset of 0, 1, 4, and 7 days using CellProfiler software (United States), and six representative morphological parameters were selected: area, perimeter, aspect ratio (shape); contrast, correlation, and entropy (texture). Box plots were used to observe the differences between the BA/OS groups on the six parameters. **(I)** ROC curves of deep learning, support vector machines (SVM), and six parameters, comparing the classification performance of four-time points.

As illustrated in Figure 3A, we first compared the POS with the mRNA expression level of two osteogenic markers, osterix (*Osx*) and runt-related transcription factor 2 (*Runx2*), on days 0, 1, 4, and 7. From the result, there were no significant differences in both *Runx2* and *Osx* gene expression from the BA/OS groups on days 0 and 1. The Osx gene expression in the OS group was upgraded on day 4 (*n* = 3, *p* < 0.05) and day 7 (*n* = 3, *p* < 0.05), and the Runx2 gene expression in the OS group was upgraded on day 7 (*n* = 3, *p* < 0.05). In comparison, the POS was significantly upregulated from day 1 (*n* = 50 cells from three biological repeats, *p* < 0.001), indicative of the early sensitivity of OCNN (Figure 3B).

Second, to scrutinize the POS at the single-cell level, we compared it with the flow cytometric analysis of the cell surface markers in the OS group on days 0, 4, and 7. On day 0, the rBMSCs had a high expression of mesenchymal stem cell marker CD90, which decreased with time; however, there were still partial overlaps with the results of day 0 (Dhaliwal et al., 2016) (Figure 3C). This decreasing trend was more evident for CD44 than CD90. Similarly, the POS also showed a right-shift tendency, similar to flow cytometry (Figure 3D).

Then, to visually inspect the POS, we compared it with the immunofluorescence staining of Runx2 at the protein level on day 4 (Figure 3E), since the spatial distribution of this nuclear transcription factor could reflect the osteogenic differentiation (Z. Chen et al., 2019). Compared with group BA, the red staining of Runx2 in the OS group was more concentrated in the nucleus, which is consistent with previous findings. Subsequently, we performed a correlation analysis of the Runx2 nuclear/cytoplasm ratio and POS (Figure 3F) and found a significant correlation (*p* < 0.05) with a moderate relationship (r = 0.4978). Based on these results, the POS is consistent with conventional biochemical markers; thus, laying the biological basis for further applications.

Single-cell morphological parameters have been widely suggested to be associated with cell phenotypes (Bakal et al., 2007; Prasad and Alizadeh, 2019; Wu et al., 2020), and some machine learning methods based on these features have been recently developed to predict osteogenesis. We extracted 25 morphological parameters (Figure 3G; Supplementary Figure S5) and selected six typical features that were significantly correlated with the cell phenotypes (Figure 3H; Supplementary Figure S6), suggesting the upregulation of the cell area, perimeter, cytoskeleton correlation, and entropy, as well as the aspect ratio and cytoskeleton contrast during osteogenesis. Support vector machine models were then developed based on these parameters.

We then compared the prediction performance between single-cell morphological parameters, support vector machine, and OCNN in a biologically independent dataset (*n* = 20) (Figure 3I). As expected, no model could distinguish cells in the two groups on day 0. Impressively, OCNN achieved a fantastic AUC of 0.998 on day 1, which was higher than that of the support vector machine (AUC = 0.807) and single-cell morphological parameters (AUCs ranged from 0.579 to 0.775). The advantage of the OCNN was maintained at day 4 (AUC = 0.993) and day 7 (AUC = 0.972) compared to those of other methods. It is worth noting that three highly correlated morphological parameters during every support vector machine training process were displayed: area, perimeter, and contrast on day 0; area, aspect ratio, and perimeter on day 1 and day 4; and area, perimeter, and entropy on day 7. From the results, it can be concluded that the area and perimeter are significant indicators for distinguishing cellular morphology. In addition, these results further support the idea of using deep-learning-based models in cell phenotype analysis.

### Screening of Osteogenic Small Molecule Drugs

**FIGURE 4:**
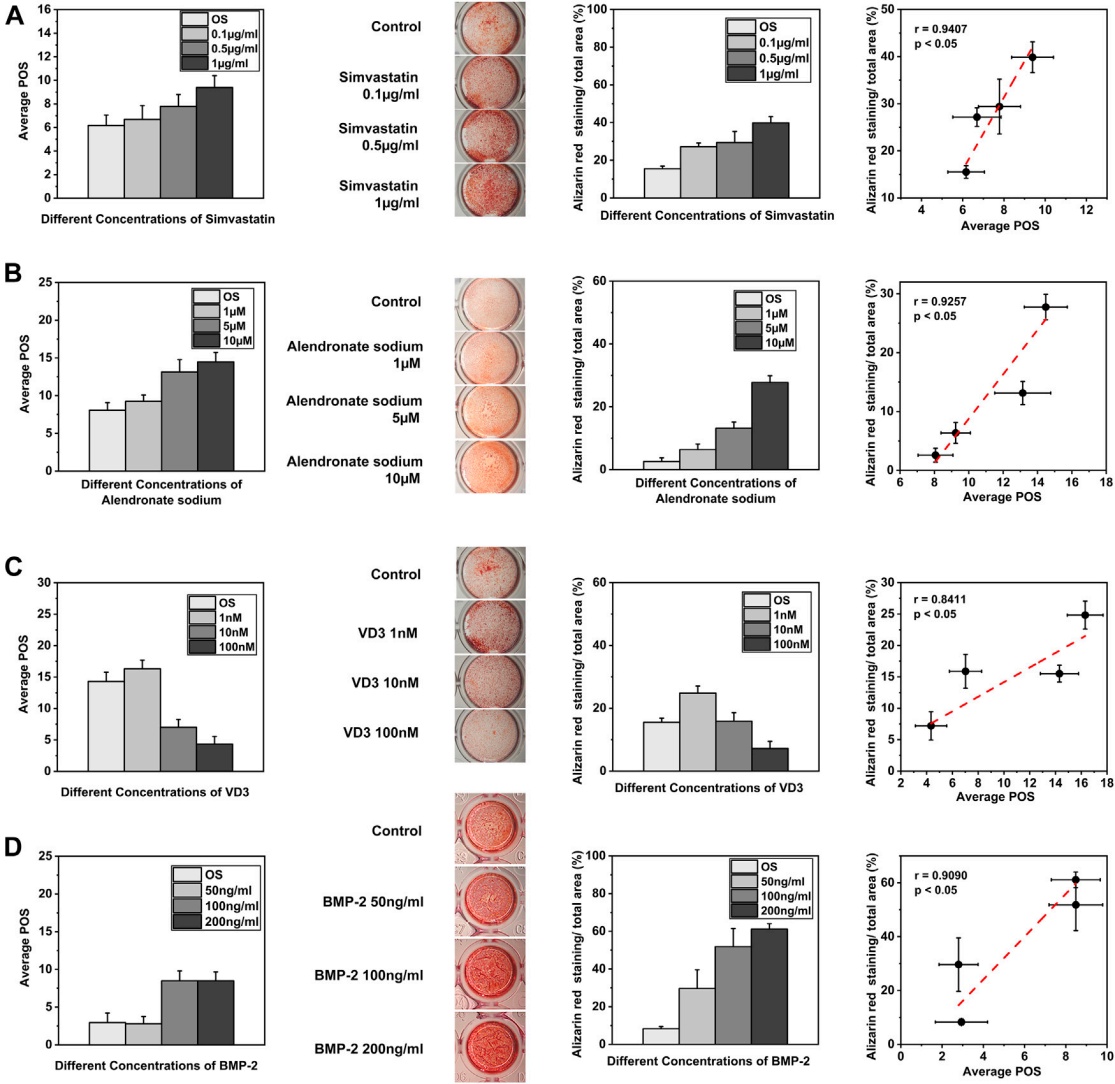
OCNN for drug screening. **(A)** The predicted osteogenic score (POS) of the rBMSCs cultured with different concentrations of simvastatin on day 1 and Alizarin red staining on day 14 was quantified by ImageJ. Linear fits were performed using the POS and Alizarin red staining areas to calculate the correlation, *p* < 0.05. **(B)** The predicted osteogenic score (POS) of the rBMSCs cultured with different concentrations of alendronate sodium on day 1 and Alizarin red staining on day 14 was quantified by ImageJ. Linear fits were performed using the POS and Alizarin red staining areas to calculate the correlation, *p*<0.05. **(C)** The predicted osteogenic score (POS) of the rBMSCs cultured with different concentrations of VD3 on day 1 and Alizarin red staining on day 14 was quantified by ImageJ. Linear fits were performed using the POS and Alizarin red staining areas to calculate the correlation, *p*<0.05. **(D)** The predicted osteogenic score (POS) of the rBMSCs cultured with different concentrations of BMP-2 on day 1 and Alizarin red staining on day 14 was quantified by ImageJ. Linear fits were performed using the POS and Alizarin red staining areas to calculate the correlation, *p*<0.05.

We examined the application potential of OCNN in predicting the osteoinduction ability of small molecule drugs or cytokines at an early stage (Figure 4). First, we examined the effects of simvastatin (Figure 4A), whose osteogenic induction ability was reported by enhancing the Rho/actin/cell rigidity pathway as well as increasing the actin filament organization and cell rigidity (Tai et al., 2015). We calculated the POS from the rBMSCs supplied with 0.1, 0.5, and 1 μg/ml simvastatin on day 1 (*n* ≥ 50), and alizarin red staining was carried out on day 14. The Pearson analysis revealed a significant correlation (*p* < 0.05) with a very strong relationship (r = 0.9407) between the OCNN prediction at the early stage (day 1) and the final osteogenesis *in vitro*.

Second, we examined the dose-dependent osteogenic induction ability of the two small molecules via other biochemical mechanisms (Figures 4B,C). Alendronate sodium affected the osteogenic differentiation by activating ERK and JNK (Fu et al., 2008), while 1α, 25-dihydroxyvitamin D3 (VD3) activated the nuclear vitamin D receptor (VDR) and promoted the osteogenic differentiation (Lou et al., 2017) (He et al., 2020). The Pearson correlation coefficient was 0.9257 (*p* < 0.05) for alendronate sodium and 0.8411 (*p* < 0.05) for 1α, 25-dihydroxyvitamin D3. These results suggested that the OCNN was able to distinguish osteogenic phenotypes modulated by different biochemical signals.

Lastly, we examined the predictive ability of the OCNN under the influence of a classic osteogenic growth factor, i.e., bone morphogenetic protein 2 (BMP-2, Figure 4D). Bone morphogenetic proteins (BMPs) are effective regulators of osteoblast proliferation and differentiation, and among them, BMP-2 has been the most studied cell growth factor in the bone tissue regeneration field (Salazar et al., 2016). The Pearson analysis revealed a significant correlation (*p* < 0.05) with a strong relationship (r = 0.9090) between the OCNN prediction and BMP-2 induced osteogenesis. Collectively, these results demonstrate the feasibility and reliability of OCNN for drug screening.

### OCNN Prediction and OCNN-Based Online Learning for Cells on Titanium Surfaces and Chemical Coatings

The substrate characteristics of biomaterials are of great importance and have a significant impact on cell morphology. Therefore, we hypothesized that the baseline OCNN may not be suitable for predicting osteogenic differentiation in this scenario. This dilemma may be tackled by the idea of online learning, which implies supplying small additional samples to the baseline model, due to the migratory nature of deep learning (Lobo et al., 2018).

**FIGURE 5:**
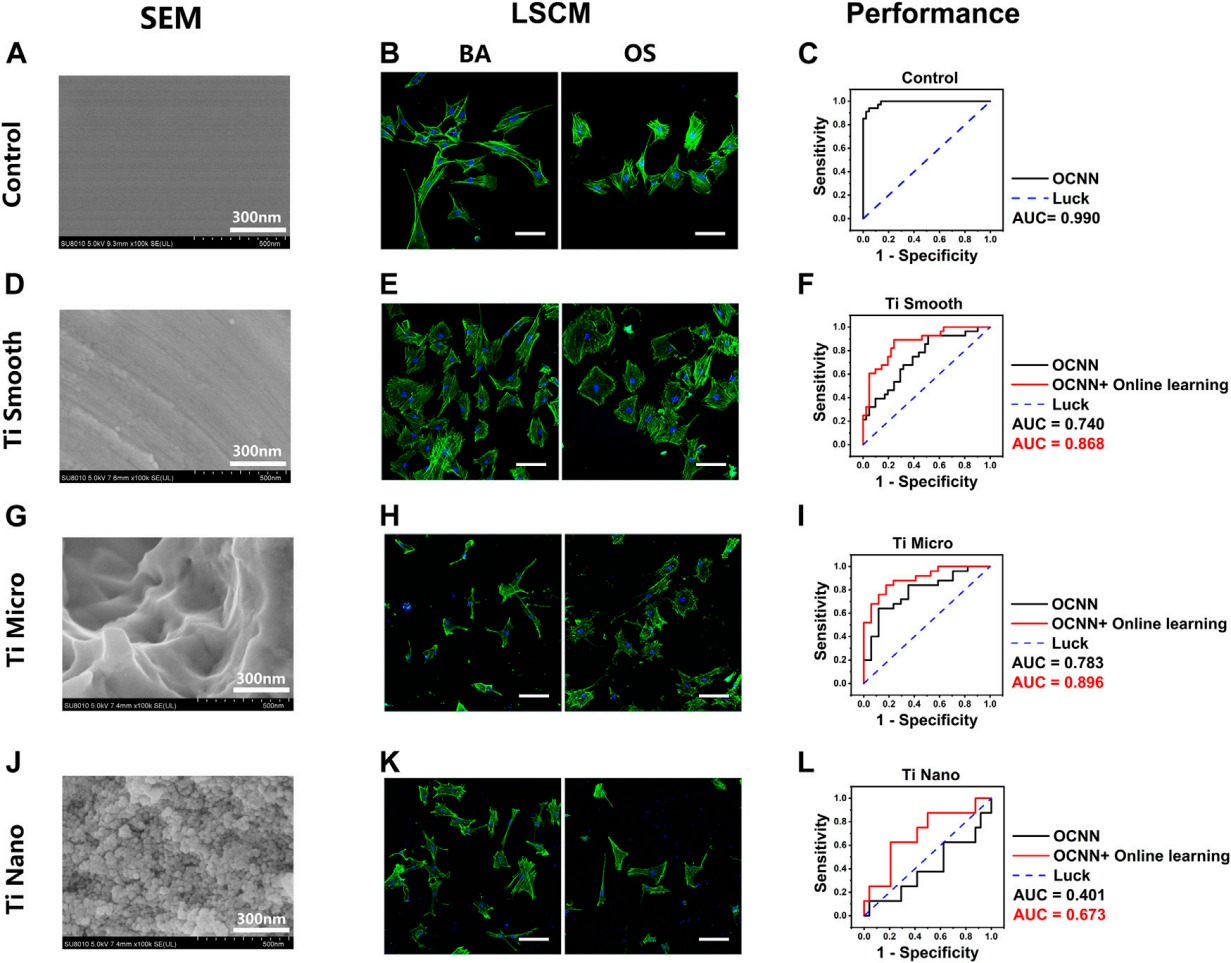
OCNN prediction and OCNN-based online learning for rBMSCs on titanium surfaces. **(A) (D) (G) (J)** SEM images of control and three different titanium surfaces: smooth, micro, and nano, scale bar = 300 nm. **(B) (E) (H) (K)** LSCM images of the rBMSCs on control and three different titanium surfaces: smooth, micro, and nano. Basal medium (BA), osteogenic supplement medium (OS). Induction time: 24 h; green: F-actin; blue: nuclei. Scale bar, 100 µm. **(C) (F) (I) (L)** Comparison of OCNN prediction and OCNN-based online learning results using ROC curves. Black line: OCNN prediction; red line: OCNN accompanied with online learning.

To examine this hypothesis, we fabricated titanium surfaces with different nanotopographies: smooth, micro, and nano, as described in our previous work. Glass coverslips were chosen as the control and were flat with no extra features under SEM (Figure 5A). On day 1, the cell morphology in the OS group exhibited no visible changes to the naked eye (Figure 5B). However, the baseline OCNN model still captured certain underlying patterns; thus, achieving a satisfactory AUC of 0.990 (Figure 5C).

For titanium surfaces, the smooth group (Figure 5D) is flat with no obvious ridges or nanoscale features, while the micro group (Figure 5G) showed numerous ridges and grooves, and the nano group (Figure 5J) showed dense nano-features. On day 1, the cell morphology on these substrates showed extreme inconsistency and diversity; thus, no patterns in group BA/OS could be observed with the naked eyes (Figures 5E,H,K). Interestingly, the baseline OCNN also performed poorly in predictions on titanium surfaces, with AUCs of 0.740 (smooth, Figure 5F), 0.783 (micro, Figure 5I), and 0.401 (nano, Figure 5L). Fortunately, after supplying small data (*n* = 50–100 and 1/16–1/8 of baseline training data) to the baseline OCNN, the AUC increased to 0.868 (smooth), 0.896 (micro), and 0.673 (nano).

**FIGURE 6:**
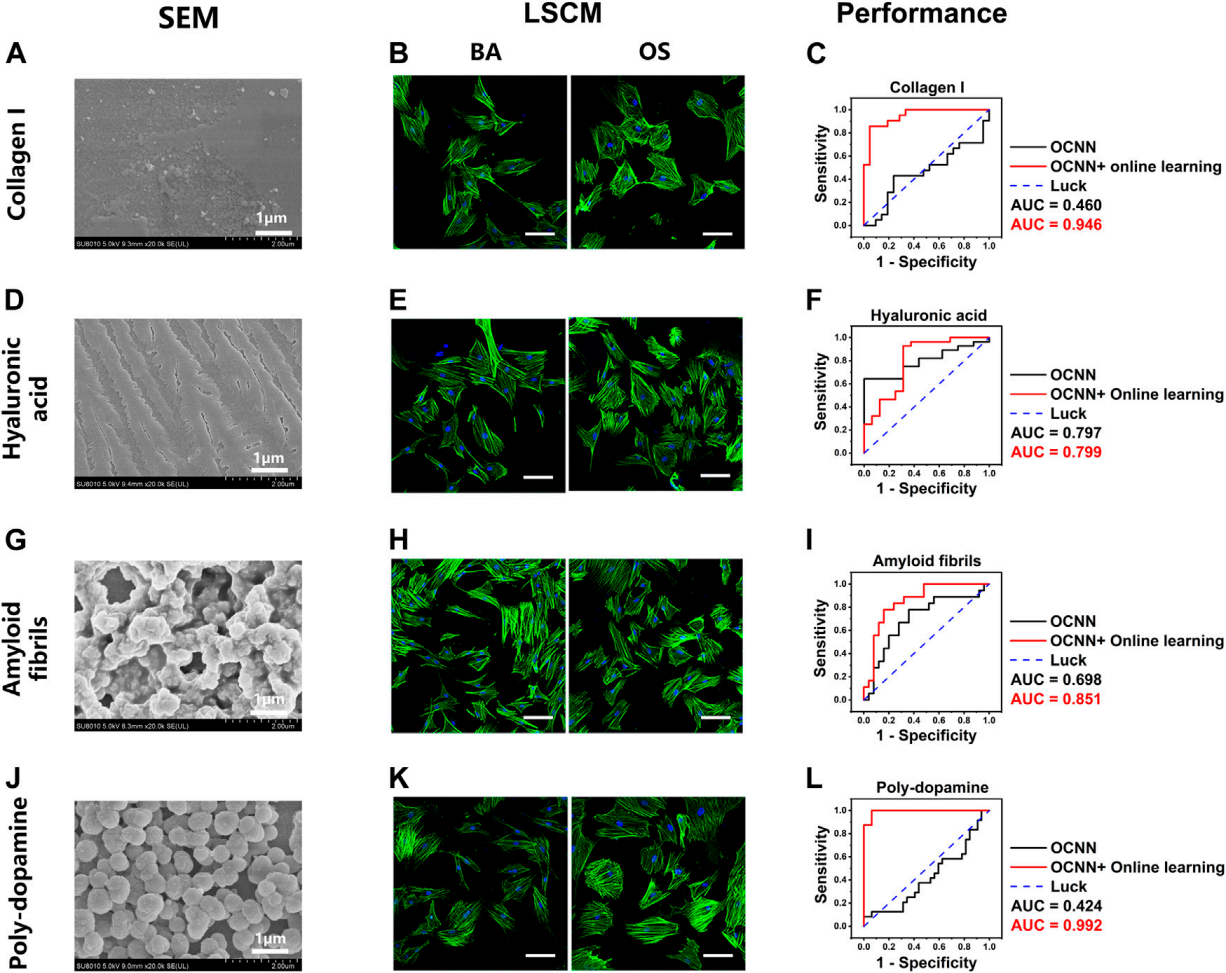
OCNN prediction and OCNN-based online learning for rBMSCs on chemical coatings. **(A) (D) (G) (J)** SEM images of four different chemical coatings: collagen I, hyaluronic acid, amyloid fibrils, and poly-dopamine, scale bar = 1 µm. **(B) (E) (H) (K)** LSCM images of the rBMSCs on four different chemical coatings: collagen I, hyaluronic acid, amyloid fibrils, and poly-dopamine. Basal medium (BA), osteogenic supplement medium (OS). Induction time: 24 h; green: F-actin; blue: nuclei. Scale bar, 100 µm. **(C) (F) (I) (L)** Comparison of the OCNN prediction and OCNN-based online learning results using ROC curves. Black line: OCNN prediction and red line: OCNN accompanied with online learning.

Next, we examined the performance of the baseline and online OCNNs on several common chemical coatings, including collagen I (Col-I), hyaluronic acid (HA), amyloid fibrils, and poly-dopamine (DOPA). On Col-I (Figure 6A) and HA (Figure 6D) coatings, no micro or nanostructures were observed under SEM, similar to the glass coverslips. Under LSCM, the cell morphology in group BA/OS could not be distinguished by the naked eye at day 1 (Figures 6B,E). However, unlike on the glass coverslips, the baseline OCNN could not predict the phenotypes associated with the OS on Col-I (AUC = 0.460) but can moderately predict on HA (AUC = 0.797), suggesting that the chemical components of substrates have an essential influence on cell phenotypes (Arora et al., 2020). After supplying online data, the AUC increased to 0.946 (Col-I, Figure 6C) and 0.799 (HA, Figure 6F). This result demonstrates the ability of online OCNN to distinguish cells cultured in microenvironments with different chemical cues.

Amyloid fibrils and DOPA coatings exhibited microspheres with diverse shapes and continuity (Figures 6G,J); thus, representing a mixture of different nanotopographies and chemical components. Under LSCM, the cell morphology in group BA/OS could not be distinguished with the naked eyes on day 1 (Figures 6H,K). The predictions of the baseline OCNN were acceptable in the amyloid fibrils (AUC = 0.698, Figure 6I) but performed poorly in DOPA (AUC = 0.424, Figure 6L). These results further suggest that the material properties of the substrates, including the nanotopography and chemical components, can substantially reform the cell morphology, which may explain the controversial association between certain cell phenotypes and final cell fate (Arora et al., 2020). After supplying online data, the AUC increased drastically to 0.851 (amyloid fibrils) and 0.992 (DOPA).

## Discussion

In this paper, we show that the CNNs can be trained with images taken by a laser confocal microscope and then classify images with slight morphological variations correctly. We applied three classical deep learning CNNs VGG-16, Inception V3, and ResNet-50 for transfer learning and achieved accuracy higher than 80% on the validation set except for day 0. Then the selected OCNN models from above also achieved excellent results on the independent test set. We believe that several conditions allowed us to achieve such high accuracy with the trained neural networks. First, we set the cell seeding at a suitable density for the study. Second, the starting size of the single-cell images is a uniform 640*640 pixels, which ensured the size and uniformity of the original input data. Third, by certain image pre-processing methods, for example, rotation, mirror flip, and scale, the number of images provided to CNN was increased and the training effect gained better than that of images without image processing.

We validated the prediction results of OCNN by conventional biochemical markers to verify the osteogenic differentiation phenotypes of rBMSCs at the corresponding training time points and there was a good fit between them, which laid the foundation for the subsequent application of OCNNs. In addition, the feature extraction of single-cell images was performed by Cellprofiler, and the classical shape and material parameters were selected to distinguish the single-cell phenotypes at uniform time points. Also, the SVM models that integrated these parameters were applied simultaneously. Neither single-cell parameters nor SVM models could reach the classification accuracy of OCNNs, reflecting the superiority of OCNNs.

For further application of OCNNs, we conducted dose-relevant predictions of osteogenic drugs and online learning-based predictions for cells cultured on different material surfaces, both of which yielded well results. In the drug screening part, we examined the ability of OCNN in predicting the osteoinduction ability of small molecule drugs or cytokines at an early stage and the results were in high consistency with 14-days alizarin red staining results. On OCNN-based online learning for cells on material surfaces, it can be seen that by online tuning the base model with a small amount of data, better prediction effects can be obtained, overcoming the phenotypical alterations on substrates with different nanotopographies and chemical components.

Compared with previous studies, this study presents several advances. First, previous relative studies adopted some machine learning techniques like linear discriminant analysis (Marklein et al., 2016), unsupervised clustering (Khayal et al., 2018), and bayesian linear regression (Cutiongco et al., 2020), to trace the osteogenic differentiation of stem cells, requiring considerable human efforts to select features and construct models, which are avoided by applying deep learning methods. Second, when we focus on image-based deep learning in the biomedical area, many other deep learning methods like YOLO (Li et al., 2021), GANs (Rubin et al., 2019; Sirinukunwattana et al., 2021) were applied and achieved good results. Compared with those methods, we conducted the evaluations of more parameters (Accuracy, Sensitivity, Specificity, Precision, F1-score, AUC) with satisfactory results. Furthermore, the strong correlation between OCNN’s predictions and osteogenic biomarkers was reached, and applications in osteogenic drug and biomaterial screening were implemented by OCNN and OCNN-based online learning. Third, cell morphology showed distinct changes and extensive versatility on substrates with different topography and chemical components. Moreover, conventional approaches usually need to train different models to associate cell phenotypes with microenvironment cues, which demands a large amount of data. By applying online learning techniques to OCNN, deep learning models can drastically improve their performance with a small amount of additional data.

Nonetheless, this study has several limitations. First, only the 2D cellular morphological characteristics were studied. Some 3D characteristics, such as cell volume and cell sphericity, are tightly correlated with 3D microenvironment cues and may have a substantial impact on the fate of cells (Bao et al., 2019; Remuzzi et al., 2020). Second, despite the cytoskeleton, more features such as nuclear skeleton, nuclear transcription factors, and chromatin morphology are also related to osteogenic differentiation. Multicolor immunofluorescent staining can capture more of this high-dimensional information simultaneously; thus, improving the prediction accuracy and generalizability. Prospectively, for future OCNN development on 3D and high-throughput data, advanced network structures and cloud-based techniques need to be constructed to tackle the increased computational complexity and the consumption of computing power. Last, in the original data acquisition process, automatic single-cell identification and segmentation procedures were lacking. In future studies, an automatic technique shall be carried out to accelerate the whole process by some algorithms like the watershed method (Ng et al., 2006), YOLO (Redmon et al., 2016), or U-net (Ronneberger et al., 2015).

In conclusion, to predict the osteogenic differentiation of rBMSCs, a deep learning model, OCNN, was successfully developed based on single-cell LSCM images. The output of the OCNN and POSs correlated well with conventional biomarkers. OCNN showed better predictions than single morphological parameters and support vector machines. It successfully predicted the dose-dependent osteogenic effects of three small molecule drugs (simvastatin, alendronate sodium, and 1α, 25-dihydroxyvitamin D3) and the osteogenic cytokine BMP-2. Moreover, OCNN with online learning successfully predicted the phenotypes associated with osteogenic differentiation on different biomaterial substrates. Therefore, this study preliminarily proved the application value and promising prospect of deep learning-based techniques in osteogenic drug screening, biomaterial development for bone tissue engineering, and cell-matrix interaction research.

## Data Availability

The original contributions presented in the study are included in the article/Supplementary Material, further inquiries can be directed to the corresponding authors.
