# Supplementary material for: Morphology-Based Deep Learning Approach for Predicting Osteogenic Differentiation
Source: Front Bioeng Biotechnol. 2022 Jan 27;9:802794. doi: 10.3389/fbioe.2021.802794 (PMC8830423; doi:10.3389/fbioe.2021.802794)
Supplement: Supplementary file 1 [file DataSheet1.docx]

Supplementary Material

# Supplementary Figures and Tables

## Supplementary Figures


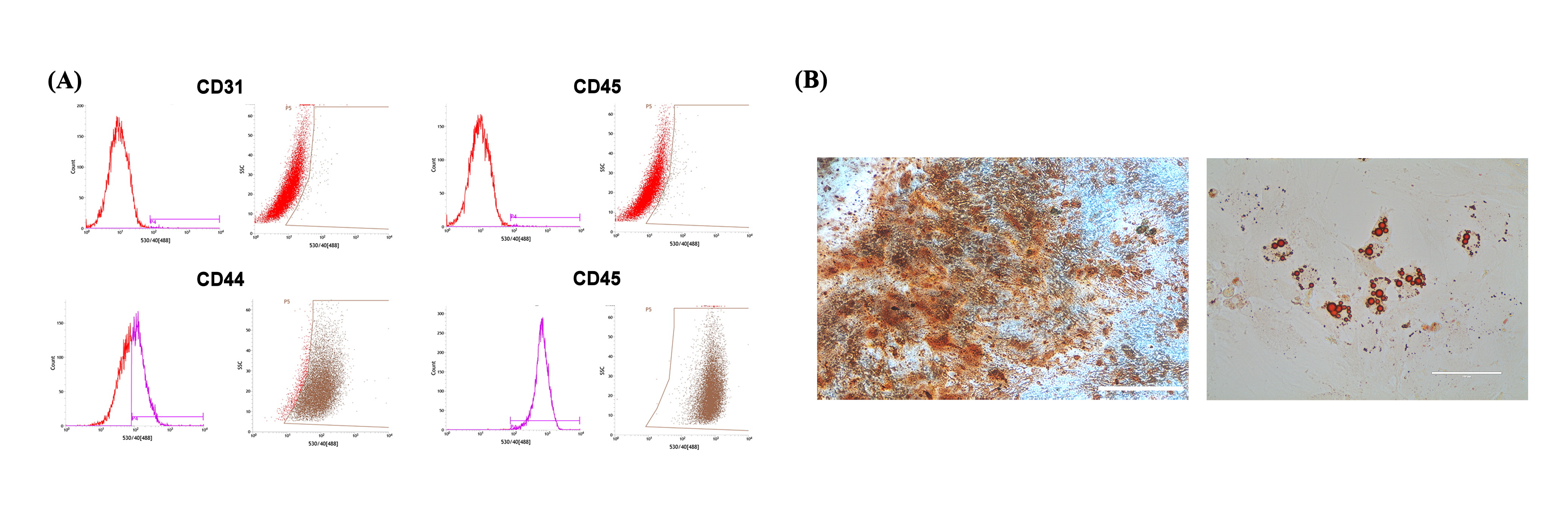


**Supplementary Figure 1.** (A) Identification of functionally relevant cell surface markers; (B) Detection of multidirectional differentiation ability of rBMSCs: left: Alizarin red staining after osteogenic differentiation, induction time:28 days, scale bar: 100µm; right: Oil red O staining after adipocyte differentiation. induction time: 21 days, scale bar: 100µm.


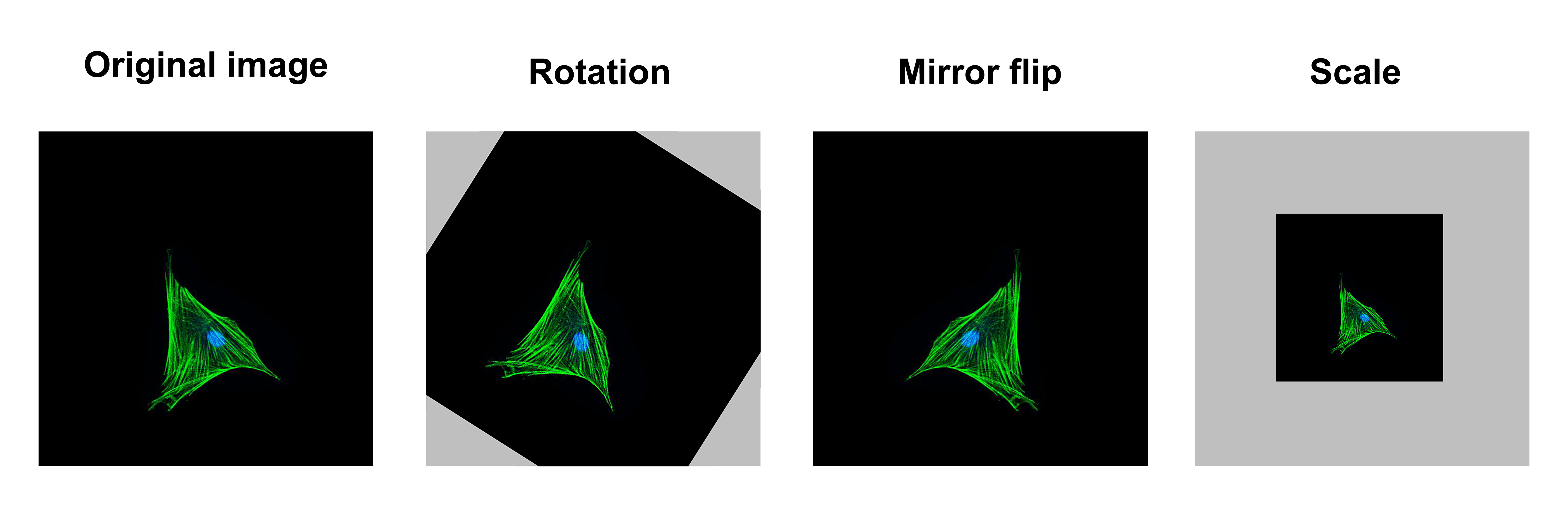


**Supplementary Figure 2.** Examples of data augmentation after rotation, mirror flip, and scale, the image was selected from the BA group on day 1.


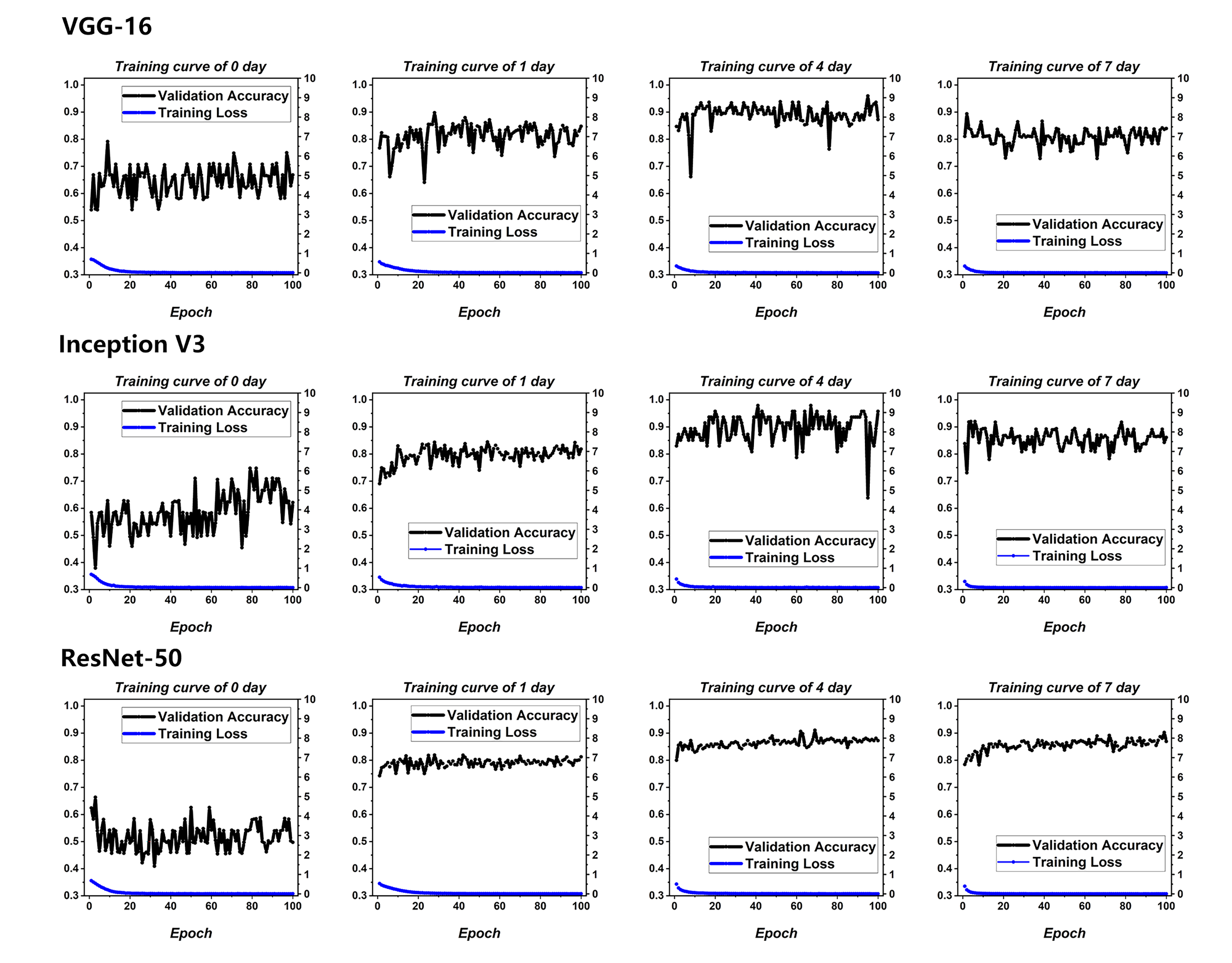


**Supplementary Figure 3.** A group of laser confocal microscopy images of mesenchymal stem cells cultured in basal (BA) and osteogenic (OS) medium for 0, 1, 4, and 7 days were trained by VGG 16, Inception V3, and ResNet-50. The validation accuracy and training loss were presented.


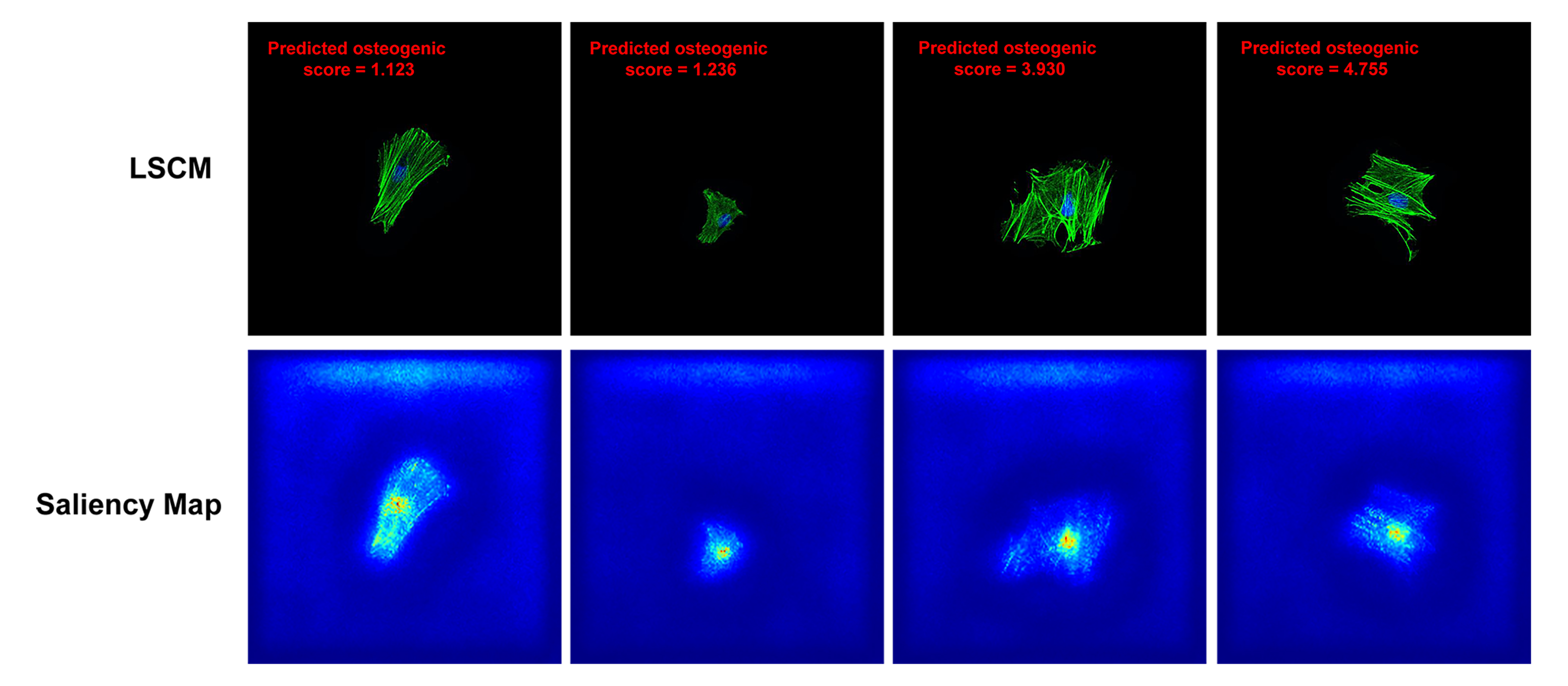


**Supplementary Figure 4.** Examples of misclassified false-positive images (Predicted osteogenic score ≥ 0) and their corresponding saliency maps. Dataset: day 1 dataset of BA group.


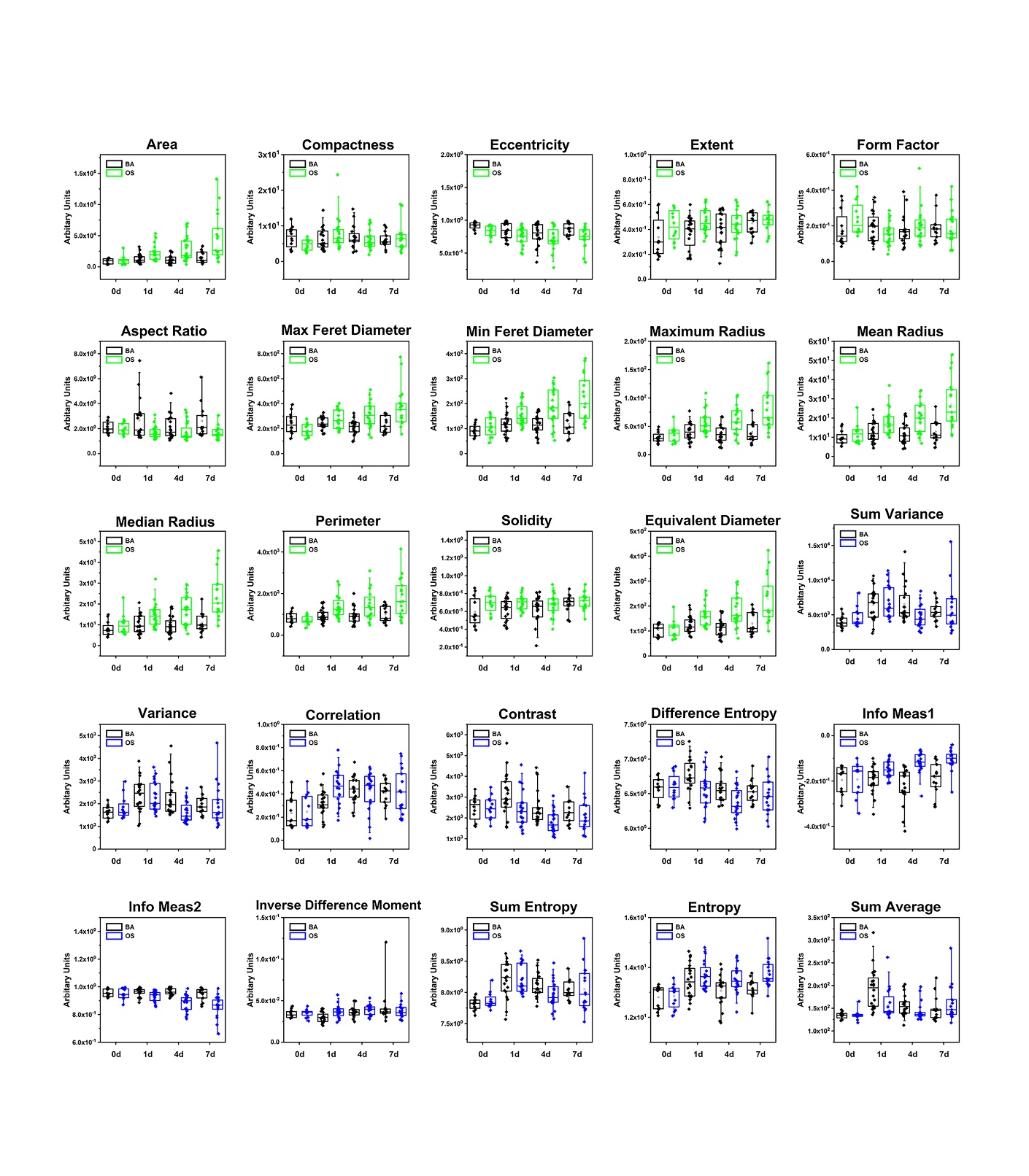


**Supplementary Figure 5.** Twenty-five cell morphological parameters were measured on the cell images of the validation dataset of 0, 1, 4, and 7 days using Cellprofiler software. Green (shape parameters); blue (texture parameters). N = 20-30 from 3 biological repeats.


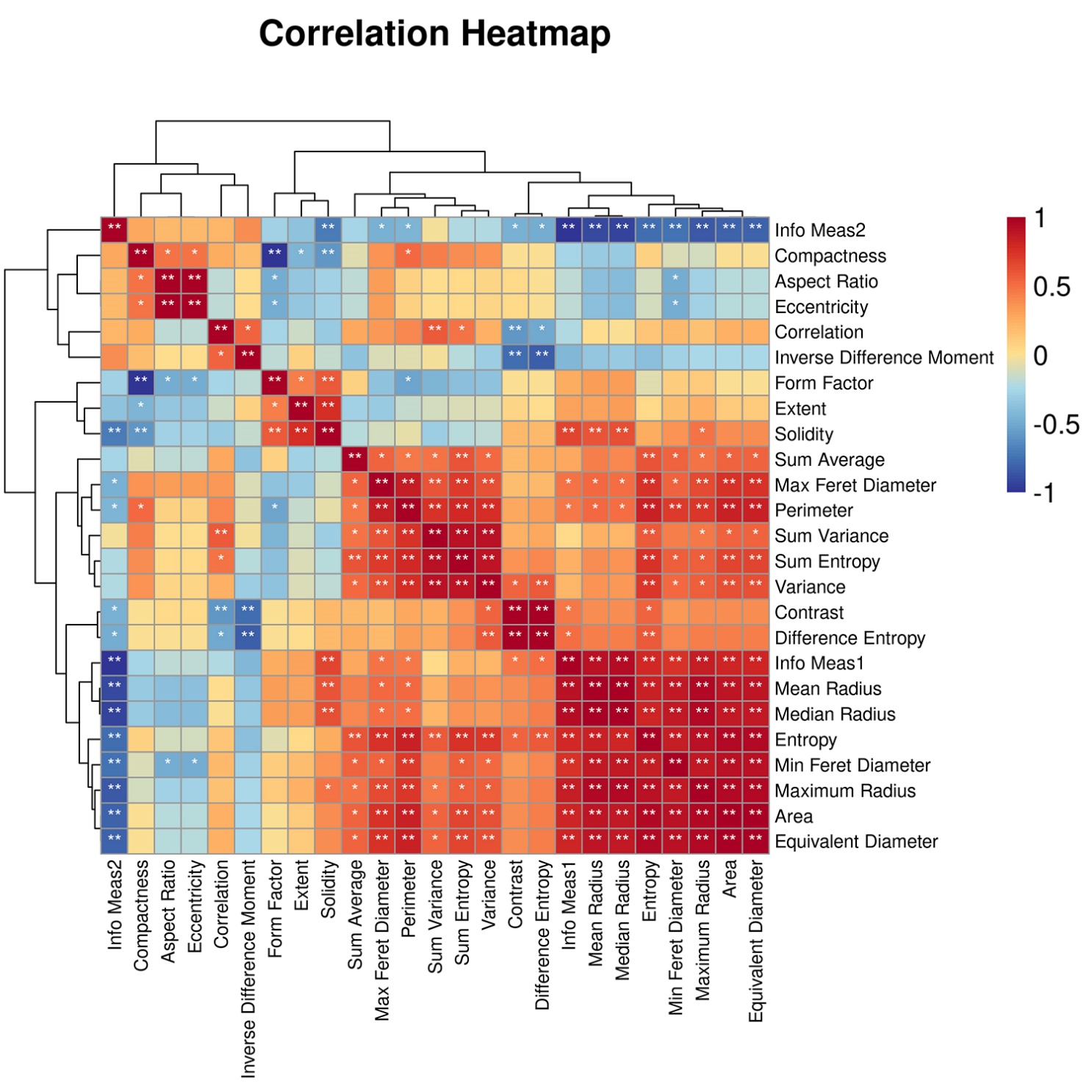


**Supplementary Figure 6.** A clustering correlation heatmap with signs was performed. The correlations between the morphological parameters were calculated with the Spearman correlation coefficient.

## Supplementary Tables

**Supplementary Table 1: Primer sequences**

| Target Gene | Forward primer sequence (5’-3’)  Reverse primer sequence (3’-5’) | Product size(bp) |
| --- | --- | --- |
| *Runx2* | CGGGAACCAAGAAGGCACA  GCCCTTGGTTCTTCCGTGT | 19  18 |
| *Osx* | GCCTACTTACCCGTCTGACTTTG  CGGATGAATGGGCAGACTGAAAC | 23  24 |
| *GADPH* | AAGTTCAACGGCACAGTCAAGG  TTCAAGTTGCCGTGTCAGTTCC | 22  22 |

*Runx 2*: Runt-related transcription factor 2. *OSX*: Osterix. *GADPH*: glyceraldehyde 3‐phosphate dehydrogenase.

**Supplementary Table 2: Basic facts of three Convolutional neural networks**

| Pre-trained CNNs | layers | Parameters(Millions) | Image Size |
| --- | --- | --- | --- |
| VGG 16 | 16 | 138 | 224*224*3 |
| ResNet-50 | 50 | 22.6 | 224*224*3 |
| Inception V3 | 46 | 23.9 | 299*299*3 |

**Supplementary Table 3: Optimization of Convolutional neural networks choice.**

Laser confocal microscopy images of mesenchymal stem cells cultured in basal (BA) and osteogenic (OS) medium for 0, 1, 4, and 7 days were trained and cross-validated in three mainstream deep learning models to choose the best model for the current study. This process used 2000 images, approximately 200-300 images at each time point, and in each medium. Each row presents five classical metrics to evaluate the classification ability of the model to distinguish BA from OS at the same time point.

| **Models** | **Metrics (10-fold cross-validation)** | | | | | |
| --- | --- | --- | --- | --- | --- | --- |
|  | **Accuracy** | **Sensitivity** | **Specificity** | **Precision** | **F1-score** | **AUC** |
| **Vgg16** | | | | | | |
| BA/OS 0d | 0.57±0.14 | 0.42±0.15 | 0.45±0.15 | 0.43±0.16 | 0.42±0.15 | 0.62±0.14 |
| BA/OS 1d | 0.81±0.03 | 0.80±0.11 | 0.79±0.04 | 0.74±0.02 | 0.77±0.05 | 0.87±0.05 |
| BA/OS 4d | 0.83±0.01 | 0.77±0.13 | 0.88±0.09 | 0.88±0.07 | 0.81±0.07 | 0.90±0.04 |
| BA/OS 7d | 0.83±0.01 | 0.77±0.16 | 0.86±0.05 | 0.87±0.03 | 0.81±0.09 | 0.86±0.07 |
| **Inception V3** | | | | | | |
| BA/OS 0d | 0.55±0.12 | 0.58±0.21 | 0.48±0.12 | 0.52±0.12 | 0.54±0.14 | 0.55±0.16 |
| BA/OS 1d | 0.85±0.05 | 0.88±0.05 | 0.83±0.10 | 0.81±0.06 | 0.85±0.06 | 0.94±0.04 |
| BA/OS 4d | 0.86±0.08 | 0.89±0.08 | 0.82±0.09 | 0.86±0.06 | 0.87±0.05 | 0.95±0.04 |
| BA/OS 7d | 0.88±0.05 | 0.95±0.04 | 0.78±0.12 | 0.86±0.07 | 0.90±0.04 | 0.97±0.02 |
| **ResNet-50** | | | | | | |
| BA/OS 0d | 0.50±0.17 | 0.60±0.15 | 0.48±0.21 | 0.59±0.13 | 0.58±0.10 | 0.48±0.17 |
| BA/OS 1d | 0.84±0.02 | 0.90±0.05 | 0.78±0.05 | 0.77±0.03 | 0.83±0.02 | 0.93±0.02 |
| BA/OS 4d | 0.85±0.02 | 0.91±0.04 | 0.78±0.04 | 0.83±0.04 | 0.87±0.02 | 0.94±0.02 |
| BA/OS 7d | 0.89±0.04 | 0.97±0.04 | 0.79±0.10 | 0.86±0.06 | 0.91±0.02 | 0.96±0.03 |
